# Resistance signatures manifested in early drug response across cancer types and species

**DOI:** 10.20517/cdr.2025.112

**Published:** 2025-08-26

**Authors:** Cole Ruoff, Allison Mitchell, Priya Mondal, Vishaka Gopalan, Arashdeep Singh, Michael Gottesman, Sridhar Hannenhalli

**Affiliations:** ^1^Cancer Data Science Lab, Center for Cancer Research, National Cancer Institute, National Institutes of Health, Bethesda, MD 20892, USA.; ^2^Laboratory of Cell Biology, Center for Cancer Research, National Cancer Institute, National Institutes of Health, Bethesda, MD 20892, USA.

**Keywords:** Cancer, resistance, epigenetics, transcription, CRISPR, evolution, therapy response

## Abstract

**Aim:** Growing evidence points to non-genetic mechanisms underlying long-term resistance to cancer therapies. These mechanisms involve pre-existing or therapy-induced transcriptional cell states that confer resistance. However, the relationship between early transcriptional responses to treatment and the eventual emergence of resistant states remains poorly understood. Furthermore, it is unclear whether such early resistance-associated transcriptional responses are evolutionarily conserved. In this study, we examine the similarity between early transcriptional responses and long-term resistant states, assess their clinical relevance, and explore their evolutionary conservation across species.

**Methods:** We integrated datasets on early drug responses and long-term resistance from multiple cancer cell lines, bacteria, and yeast to identify early transcriptional changes predictive of long-term resistance and assess their evolutionary conservation. Using genome-wide CRISPR-Cas9 knockout screens, we evaluated the impact of genes associated with resistant transcriptional states on drug sensitivity. Clinical datasets were analyzed to explore the prognostic value of the identified resistance-associated gene signatures.

**Results:** We found that transcriptional states observed in drug-naive cells and shortly after treatment overlapped with those seen in fully resistant populations. Some of these shared features appear to be evolutionarily conserved. Knockout of genes marking resistant states sensitized ovarian cancer cells to Prexasertib. Moreover, early resistance gene signatures effectively distinguished therapy responders from non-responders in multiple clinical cancer trials and differentiated premalignant breast lesions that progressed to malignancy from those that remained benign.

**Conclusion:** Early cellular transcriptional responses to therapy exhibit key similarities to fully resistant states across different drugs, cancer types, and species. Gene signatures defining these early resistance states have prognostic value in clinical settings.

## INTRODUCTION

Although numerous chemotherapeutic, targeted, and monoclonal antibody-based drugs have been approved for various cancers, resistance invariably emerges, resulting in relapse and, ultimately, patient death. In fact, therapeutic resistance remains the leading cause of treatment failure and cancer-related mortality^[1]^. Several mechanisms underlying resistance have been identified^[2]^. While mutation-driven resistance has traditionally been the predominant paradigm^[3]^, recent evidence highlights an epigenetic or transcriptional basis for resistance. In this model, resistance arises from a subpopulation of malignant cells that adopt drug-resistant transcriptional states^[4,5]^. These pre-existing, reversible resistant cells - referred to as drug-tolerant persister (DTP) cells^[6]^ - are distinct from stable, emergent resistant states^[7]^.

Recent studies have begun to characterize these resistant transcriptional states^[8,9]^, but several key questions remain. For instance, it is unclear whether, and to what extent, long-term transcriptional resistance is an inherent cellular response that becomes evident shortly after drug exposure. Additionally, the extent to which early cellular responses are shared across cancer types and drugs - and whether these responses are evolutionarily conserved - remains unknown^[10]^. Identifying such broadly conserved early responses may hold clinical utility. These key questions form the basis of our investigation.

To address these questions, we first derived a broad, long-term resistance signature from previously published studies involving multiple cell lines and drugs^[9]^. We then analyzed single-cell transcriptomic data capturing the early transcriptional response to drug treatment^[11]^. By comparing these early response scRNA-seq data with the long-term resistance-associated transcriptional signature, we identified features of long-term resistance that are already apparent in early response states. Furthermore, we assessed the evolutionary conservation of these early resistance-associated responses using previously published transcriptional datasets from the bacterium *Escherichia coli* (*E. coli*)^[12]^ and the fungus *Candida auris* (*C. auris*)^[13]^.

Our findings indicate that transcriptional states associated with long-term resistance are already evident shortly after drug exposure. Across all three cell lines analyzed, we observed early transcriptional responses that closely resembled long-term resistant states. Notably, our analyses also suggest that these resistance-associated states may pre-exist prior to treatment, in addition to being induced by drug exposure. These early and pre-existing states share common features across cell lines, including elevated oxidative phosphorylation, epithelial-mesenchymal transition (EMT), hypoxia signaling, and MYC pathway activity. Interestingly, similar transcriptional states were observed in *E. coli* and *C. auris* following exposure to various drugs, suggesting that this mode of resistance may represent an evolutionarily conserved mechanism. Using a genome-wide CRISPR screen, we demonstrated that knocking out markers of the early resistance signature increases drug sensitivity. Finally, we show that these resistance-associated early transcriptional signatures have prognostic relevance across multiple cancer types and clinical contexts. Specifically, they can distinguish responders from non-responders and differentiate premalignant breast lesions that progress to malignancy from those that remain benign.

## METHODS

### Creating the resistance signature

We developed a 72-gene drug resistance gene signature by aggregating ranked gene lists from previously conducted drug treatment experiments in six cell lines (COLO829, A375, HT29, MMACSF, EFM192A, and BT474)^[9]^. These cell lines were exposed to drugs for 10 days, after which the surviving cells were sequenced. For each cell line, we identified differentially expressed genes between day 10 and day 0 using the FindAllMarkers() function from Seurat (v5.2.1)^[14]^. Genes that were significantly upregulated (log_2_FC > 0, adjusted *P* < 0.05) were selected and ranked by log_2_FC. We then used Robust Rank Aggregation (RRA; v1.2.1)^[15]^ to generate a single ranked list and retained 72 genes with an adjusted rank *P*-value < 0.05.

To validate this signature, we used time-course data from drug resistance development in PC9 lung cancer cells treated with Osimertinib^[9]^, with single-cell transcriptomics conducted at days 0, 3, 7, and 14. At each time point, fold changes for each gene were computed relative to all other time points using Seurat’s FoldChange() function. Enrichment of the 72-gene resistance signature was then evaluated at each time point using Gene Set Enrichment Analysis (GSEA) via the fgsea R package (v1.32.4)^[16]^.

### Single-cell dataset processing

For the Oren *et al*. dataset^[9]^, raw count matrices were downloaded from the public repository cited in the original publication (GEO: GSE150949). Cells with fewer than 200 or more than 2,500 detected features were excluded. Data were normalized via log transformation using a scale factor of 10,000.

For the Srivatsan *et al*. dataset^[11]^, filtered count matrices were downloaded from GEO (GSE139944). Genes expressed in fewer than 100 cells were removed to retain biologically relevant genes. Normalization was performed as above. Pre- and post-treatment cells were processed identically to minimize batch effects.

### Functional enrichment analysis

Pathway enrichment analysis was conducted using the clusterProfiler R package (v4.14.6)^[17]^, including pathways from MSigDB^[18]^ hallmarks, intratumoral heterogeneity meta-programs (ITH MPs)^[19]^, and Gene Ontology (GO) biological processes. The enricher() function was used for hallmarks and MPs, while enrichGO() was used for GO terms. The number of overlapping genes between each pathway and the gene set of interest was reported as counts.

### AUCell threshold

For all applications of AUCell (v1.28)^[20]^, we estimated an activation threshold for a given gene signature by generating a background distribution of AUCell scores using 100 control gene sets with expression levels matched to the target genes. These control gene sets were constructed following a procedure similar to Seurat’s AddModuleScore() function.

### Odds ratio for resistance signature activity in cell clusters

We computed the odds ratio for resistance signature activity in each cell cluster using Fisher’s exact test. Clusters with an odds ratio > 1 and an adjusted *P*-value < 0.05 were defined as resistance-activated clusters (RACs). Fold enrichment for a drug class was calculated relative to the random expectation.

### Global RAC gene signatures

To define global RAC signatures, we used Seurat’s FindAllMarkers() function to compare all RAC cells to non-RAC cells within each cell line. Genes with an adjusted *P*-value < 0.05 and log_2_FC > 0 were retained. The top 200 genes, ranked by log_2_FC, were selected as the signature.

### Grouping the RACs across cell lines into superclusters

We compiled a list of 3,084 genes, representing the intersection of the top 2,000 most variable genes in each cell line. For each gene, we calculated the differential mean expression in RACs relative to other cells within each line. RACs were then hierarchically clustered based on pairwise Spearman correlation of these gene expression vectors. Superclusters were defined as groups of highly correlated RACs containing components from at least two of the three cell lines.

### Supercluster consensus signature creation

To generate a consensus signature for each supercluster, we followed the same approach as used for the initial resistance signature. For each RAC within a supercluster, we selected differentially expressed genes (log_2_FC > 0, adjusted *P* < 0.05), ranked them by log_2_FC, and aggregated the lists using RRA, retaining genes with an adjusted rank *P*-value < 0.05. The same method was applied to identify downregulated genes (log_2_FC < 0).

### Supercluster cell cycle phase plots

Cell cycle phase assignment within each supercluster was performed using Seurat’s CellCycleScoring() function, incorporating an additional G0 phase gene signature^[21]^. Each cell was assigned to the phase corresponding to its highest-scoring signature.

### Drug class-specific superclusters

To determine whether cells treated with a specific drug class were overrepresented in a supercluster, we calculated the odds ratio comparing the proportion of treated cells within the supercluster’s component clusters to the proportion outside those clusters.

### Origin and maintenance of OVCAR8 cells

The OVCAR8 cell line, derived from the ascites of a patient with progressive high-grade serous ovarian carcinoma, was obtained from the NCI Developmental Therapeutics Program (DTP). Cells were cultured in RPMI-1640 medium with 10% fetal bovine serum (FBS) and 1% penicillin-streptomycin, and maintained at 37 °C in a humidified incubator with 5% CO_2_. Mycoplasma contamination testing was performed regularly.

### CRISPR screening

A genome-wide Brunello CRISPR knockout (KO) lentiviral library (Addgene #73178, Watertown, MA, USA) was used to identify genes conferring resistance to the CHEK1/2 inhibitor Prexasertib. The library includes 76,441 gRNAs targeting 19,114 genes and 1,000 non-targeting controls. OVCAR8 cells stably expressing Cas9 (Addgene # 52962-LV) were transduced at an MOI of ~0.3, followed by puromycin selection (5 μg/mL for three days). The selected population was divided into treatment (30 nM Prexasertib) and control (DMSO) arms, each cultured for 10 generations with 500× coverage. Genomic DNA was extracted using the QIAmp DNA Blood Cell Maxi Kit (Qiagen, Germantown, MD, USA) according to the manufacturer’s protocol. The sgRNA barcodes were PCR-amplified using Taq polymerase and adapted for sequencing. The desired DNA product was purified using a 6% TBE gel (Invitrogen, Waltham, MA, USA) and the samples were sequenced on an Illumina HiSeq2000. Read counts and hit identification were performed using MAGeCK (v0.5.7)^[22]^, with gene ranking based on RRA and normalization to non-targeting gRNA distributions.

### Kaplan-Meier plots

Kaplan-Meier survival analysis was conducted using the survminer R package (v0.5.0)^[23]^. Samples were divided into high and low expression groups using the top and bottom 50th percentiles of ssGSEA scores, derived from the GSVA R package (v2.0.7)^[24]^. Survival time is shown in days, and *P*-values were calculated using the log-rank test.

### Cox regression

Cox proportional hazards regression was performed using the survival R package (v3.8-3)^[25]^. Overall survival was modeled using ssGSEA scores for the resistance signature, age, sex, and tumor purity (when available).

### Scoring recurrence, pre-malignancy, and drug response data

For recurrence, pre-malignancy, and drug response analyses, patient samples were scored using ssGSEA with the global RAC or supercluster signatures.

### Creating resistance ortholog signatures

To construct a drug resistance signature for *C. auris*, we retrieved differential gene expression data comparing resistant and sensitive populations from the original study^[13]^, selecting genes with log_2_FC > 0 and adjusted *P* < 0.05. For *E. coli*, we used expression data from nine experiments involving various antimicrobials^[12]^, identifying upregulated genes (log_2_FC > 0, adjusted *P* < 0.05) in each. Genes present in at least 5 of the 9 experiments were selected. Orthologs were mapped to the human genome using OMA Browser^[26]^.

### Scoring for resistance ortholog signatures in superclusters

Cells from early response data were scored for the *C. auris* and *E. coli* ortholog resistance signatures using AUCell. Score distributions were compared using the Wilcoxon rank-sum test.

### Functional enrichment of conserved genes

To assess the biological relevance of conserved resistance genes, we identified overlaps between the *C. auris* or *E. coli* resistance signatures and supercluster signatures with enriched AUCell scores. These overlapping genes were analyzed for enrichment in MSigDB hallmarks, ITH MPs, and GO processes as described above.

## RESULTS

### Multiple cell states in pre-treatment and early response data express long-term resistance programs

**Figure 1:**
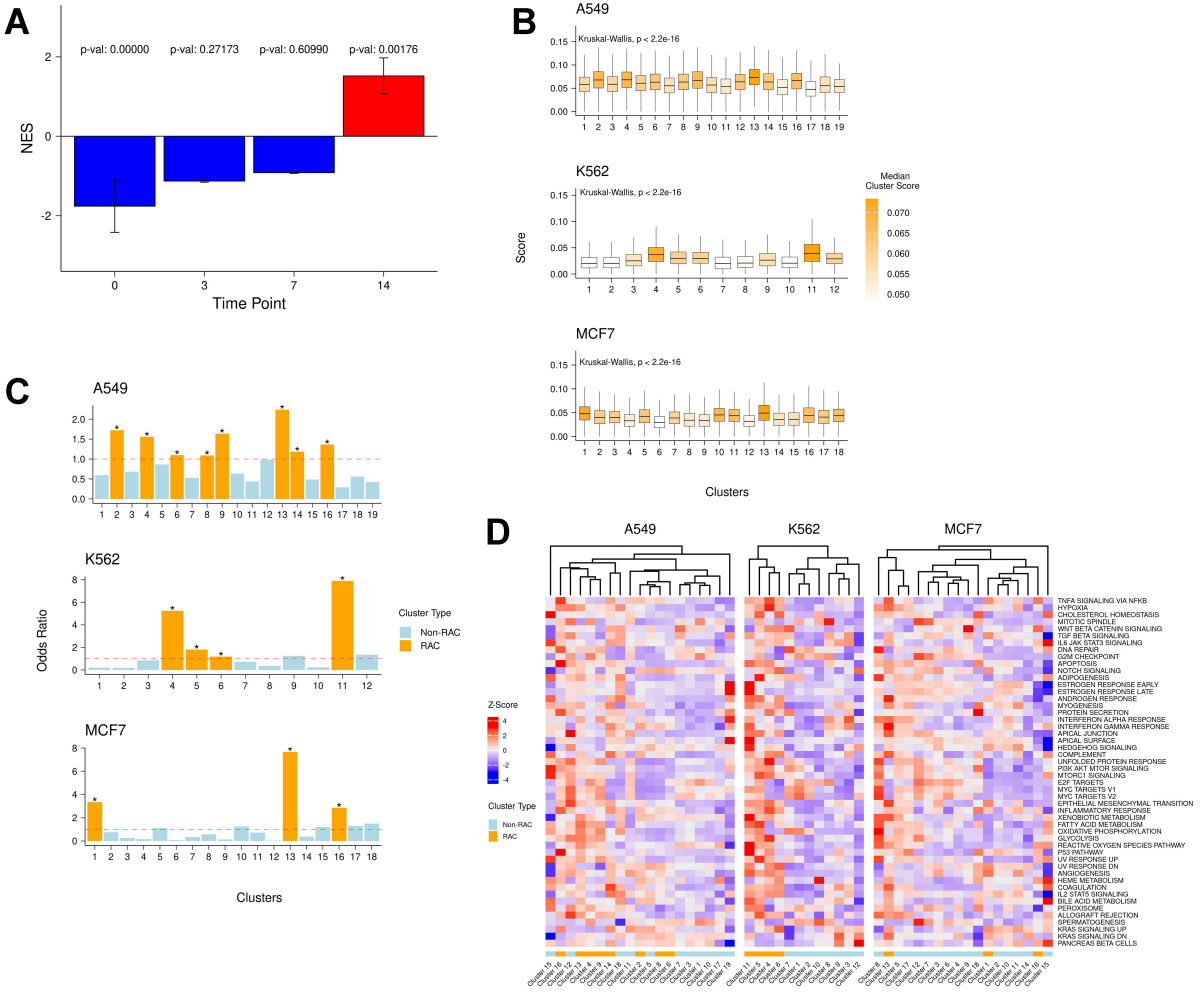
(A) Enrichment of the derived drug resistance signature gene at days 0, 3, 7, and 14 in the Oren *et al.* dataset^[9]^; (B) Distributions of raw AUCell scores of the resistance signature in each cluster across all cell lines; (C) Enrichment odds ratio of resistance-active cells in each cluster. Asterisks indicate significant odds ratio values (*P* < 0.05); (D) Standardized (z-score) mean AUCell scores of cancer hallmark pathways in each cluster across all cell lines. NES: Normalized Enrichment Score.

To identify early post-treatment manifestations of long-term drug resistance, we developed a gene signature composed of commonly upregulated genes [Supplementary Table 1] shared across six drug-resistant samples from previous studies^[9]^. These resistance studies involved a wide variety of cancer cell lines treated with different drugs, and thus, the derived consensus gene set represents a general drug resistance program. We validated this gene signature using an independent dataset that included multiple timepoints during the development of resistance in a different cancer type - lung adenocarcinoma^[9]^. Notably, we observed strong enrichment of the signature at day 14 [Figure 1A], supporting the generality of the derived resistance program. Functional enrichment analysis [Supplementary Figure 1A] revealed the involvement of known resistance-associated pathways such as EMT and hypoxia^[27,28]^.

Next, we analyzed an independent, large-scale dataset containing 581,308 cells, including both drug-naive and 24-hour post-treatment cells from three cancer cell lines (A549, K562, MCF7) treated with 188 compounds across 15 drug classes^[11]^. Notably, these three cell lines differ from the six used to derive the long-term resistance signature. For each of the three cell lines, we independently assessed the relative expression of the consensus resistance signature within each transcriptional cluster. In every cell line, certain clusters exhibited significantly higher resistance signature scores [Figure 1B], suggesting either the pre-existence of resistance-associated gene expression or its induction within 24 h of drug treatment.

To identify the cell states that may represent early manifestations of resistance, we evaluated the relative enrichment of resistance-active cells in each cluster compared to all other clusters within the same cell line [Figure 1C]. Multiple clusters in each cell line were significantly enriched for resistance-active cells, which we designated as RAC - 15 in total.

Moreover, in each cell line, some clusters exhibited high levels of resistance signature expression even before treatment, consistent with pre-existing resistance^[7]^ [Supplementary Figure 1B]. Collectively, these findings indicate that multiple cell states, both pre-treatment and within the early drug response phase, express resistance programs resembling those observed in long-term drug-resistant samples.

### RACs are enriched for conserved resistance pathways across cell lines

Having identified RACs in each cell line, we next aimed to functionally characterize them using cancer hallmark signatures (from MsigDB^[18]^) and intratumor heterogeneity meta-programs (ITH MPs)^[19]^ [Figure 1D and Supplementary Figure 1C]. In two of the three cell lines, most RACs clustered together hierarchially, although there was some diversity in hallmark activity among RACs within each line. This is consistent with previously reported distinct and functionally divergent resistant states^[8]^. Notably, in each cell line, RACs exhibited both high and low enrichment of cell cycle-related signatures, aligning with earlier observations of variability in cell cycling among resistance states^[9]^. A similar pattern emerged when we analyzed gene enrichment between all RAC and non-RAC cells in each cell line, regardless of clusters (Supplementary Materials - “Global RAC Signature Enrichment”).

**Figure 2:**
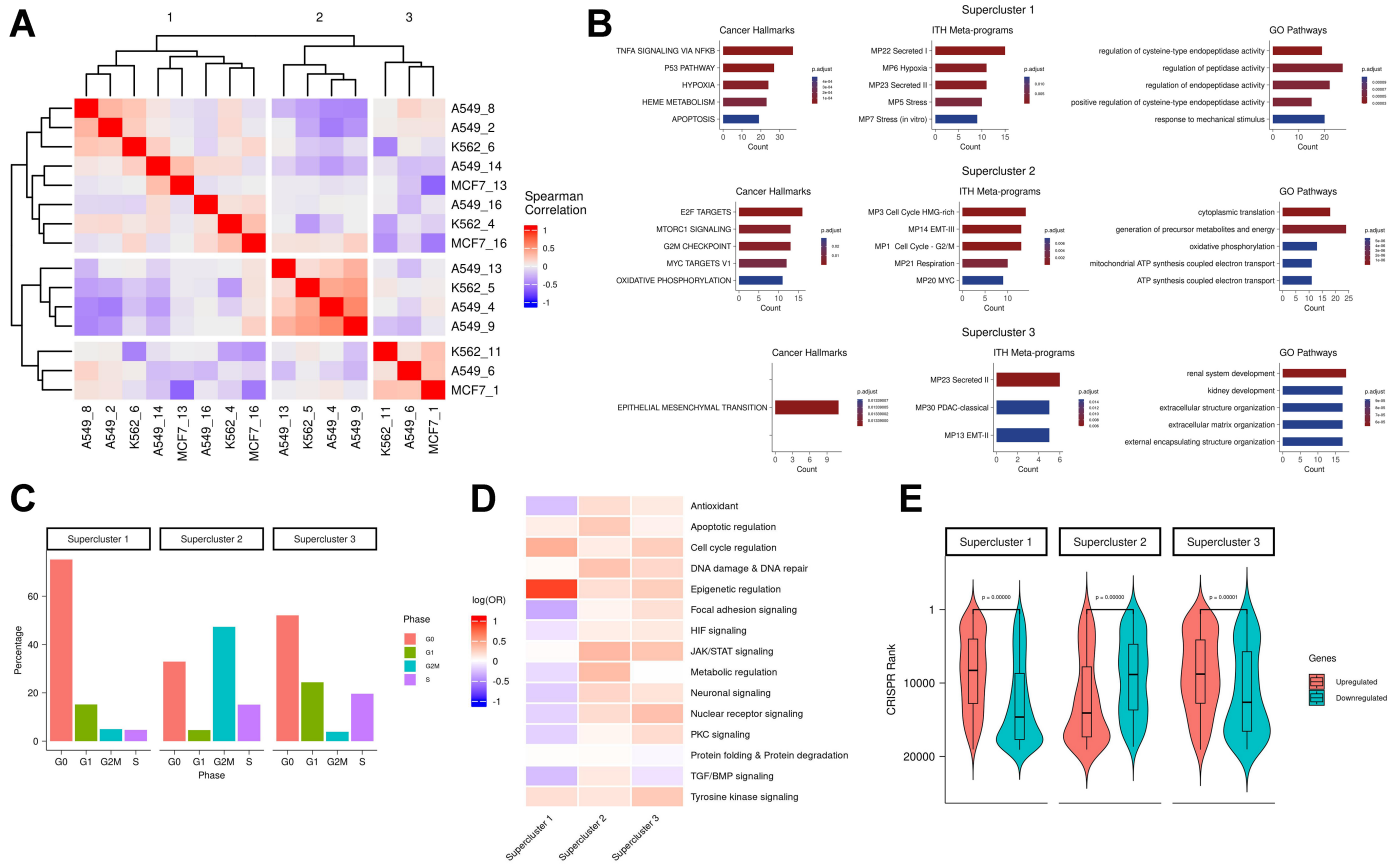
(A) All-by-all Spearman correlation matrix of RAC differential mean expression vectors; (B) Functional enrichment of supercluster signatures. Columns from left to right show enrichment in cancer hallmarks, ITH MPs, and GO pathways, respectively. Rows represent the three superclusters. Count values indicate the number of overlapping genes between the supercluster signature and the enriched pathway; (C) Distribution of cell cycle phase percentages for each supercluster (G0 = red; G1 = green; G2M = blue; S = purple); (D) Drug class enrichment for each supercluster; (E) CRISPR rank distributions (Y-axis) for upregulated (red) and downregulated (blue) genes in each supercluster. RAC: Resistance-activated cluster; ITH MPs: intratumoral heterogeneity meta-programs; GO: Gene Ontology.

To define pan-cell line resistance signatures, we grouped all 15 RACs from the three cell lines into three superclusters based on their transcriptional similarities [Figure 2A and Supplementary Figure 2A]. Each supercluster included RACs from at least two cell lines. To further probe their function, we generated two gene sets per supercluster by aggregating genes consistently upregulated or downregulated in all component RACs from each line [Supplementary Table 1].

Functional enrichment analysis of the upregulated genes in each supercluster revealed both shared and distinct enriched programs across the three superclusters [Figure 2B]. Supercluster 1 was enriched for hypoxia and TNFα signalling - pathways previously implicated in therapy resistance across various cancer types^[29]^. Hypoxia contributes directly to drug resistance by reducing drug cytotoxicity^[28]^ and promoting cellular plasticity, which enables adaptive survival responses^[30]^. Supercluster 2 was enriched for MYC activity, a key driver of drug resistance^[31,32]^, and showed metabolic rewiring^[33]^ characterized by enrichment in respiration and oxidative phosphorylation pathways. Supercluster 3 was enriched for EMT and secretion pathways, both commonly associated with resistance mechanisms^[27,34]^. Enrichment analysis of the downregulated gene signatures for each supercluster is provided in the Supplementary Materials - “Supercluster downregulated gene signature enrichment”.

Pathways enriched in the supercluster 2 signature included several related to cell cycling progression, indicating a proliferative resistant state. This was supported by notable differences in the distribution of cell cycle phases among superclusters [Figure 2C], suggesting that resistant states differ in their proliferation rates - likely due to metabolic variation^[9]^. In particular, supercluster 1 exhibited the highest proportion of cells in the G0 phase, suggestive of a quiescent drug-resistant state^[35,36]^.

To investigate the influence of drug classes on resistance states, we analyzed the association between superclusters and specific drug treatments. Supercluster 1 consisted predominantly of cells treated with drugs targeting epigenetic regulation, while superclusters 2 and 3 included cells treated with a broader range of drug classes [Figure 2D]. These patterns suggest that certain drug classes may preferentially induce specific resistance states. Our findings support the hypothesis that some drugs trigger similar resistance states across different cancer types, implying that the transcriptional response may be more dependent on the treatment than on the cancer type itself.

### CRISPR knockout of resistance-associated genes sensitizes cells to drug treatment

Here, we experimentally validated the resistance gene signatures by testing whether genes defining the superclusters have a predictable impact on drug resistance in a novel context. We selected a cancer type and drug that were not included in any of the previously analyzed datasets, and assessed whether knocking out genes characteristic of each supercluster would increase sensitivity to cytotoxic drugs. To this end, we performed a genome-wide CRISPR knockout screen in the ovarian cancer-derived cell line OVCAR8 and measured the effect of individual gene knockouts on cell viability following treatment with the CHK1/2 checkpoint kinase inhibitor Prexasertib over 10 cell generations. Genes were ranked based on the degree of increased drug sensitivity upon knockout; higher-ranking genes in this list are more likely to mediate drug resistance. Encouragingly, we found that in superclusters 1 and 3, the upregulated genes exhibited significantly higher CRISPR-based ranks than the downregulated genes (*P*-value ≈ 0 for both; Figure 2E). This finding - that upregulated genes in the low-proliferation states (superclusters 1 and 3) scored higher in the Prexasertib screen - is consistent with the drug’s known mechanism of action, namely replication catastrophe^[37]^. Conversely, in supercluster 2, which is characterized by a proliferative state, knockout of the upregulated genes led to reduced sensitivity to the drug [Figure 2E]. These results suggest that disrupting transcriptional states associated with resistance in the early response phase could offer a viable strategy for modulating drug tolerance.

### Conserved resistance states have clinical relevance

**Figure 3:**
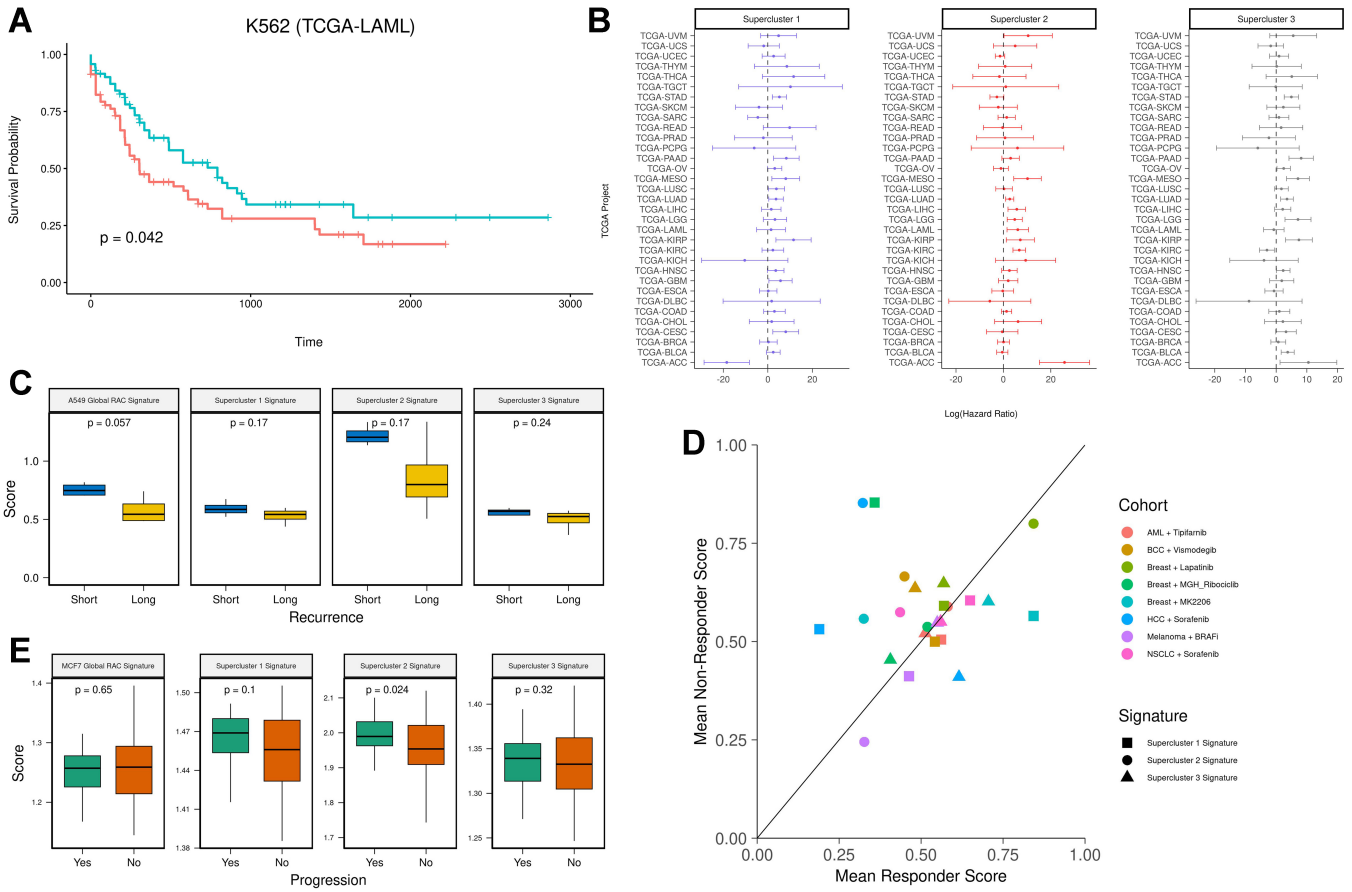
(A) Kaplan-Meier survival analysis of the K562 RAC signature in the LAML cohort. Patients were stratified into two groups based on K562 RAC signature scores (red = high, blue = low); (B) Hazard ratios with 95% confidence intervals for supercluster signatures across all 33 TCGA cancer types; (C) Pre-treatment RAC signature scores in lung cancer patients prior to tyrosine kinase inhibitor treatment. The Y-axis shows ssGSEA scores, and the X-axis stratifies patients by recurrence time (blue = short-term recurrence; yellow = long-term recurrence); (D) Mean supercluster signature scores for responders and non-responders across various treatment cohorts (cohort = drug × cancer type); (E) RAC signature scores in premalignant lesions. The Y-axis represents ssGSEA scores, and the X-axis stratifies patients by progression status (green = progression; orange = no progression). RAC: Resistance-activated cluster.

Here, we aimed to evaluate the clinical relevance of cell line-specific global RAC signatures and the RAC supercluster gene signatures. First, for each cell line, we assessed the association between the cell line’s global RAC signature and patient survival in the corresponding cancer type using data from TCGA. We scored the patient samples with the respective signature and performed Cox regression analysis on overall survival. As shown in Figure 3A, the Kaplan-Meier plot for the K562 RAC signature applied to the LAML cohort suggests that high expression of the global RAC signature is associated with poorer survival. Although the results are not statistically significant, all three cases demonstrated a positive association between the RAC signature score and hazard ratio (A549-LUAD: HR = 2.1, *P*-value = 0.15; K562-LAML: HR = 2.5, *P*-value = 0.14; MCF7-BRCA: HR = 2.0, *P*-value = 0.27).

Because superclusters represent multiple cancer types, we repeated the above analysis using the supercluster signatures across all TCGA cohorts [Figure 3B]. Despite considerable variability across cancer types, the distribution of log-transformed hazard ratios was significantly greater than zero (*P*-values = 0.0176, 0.0016, and 0.0082, respectively, for the three cell lines; one-sample *t-*test).

Next, to evaluate the predictive value of the supercluster signatures for therapy response, we analyzed a dataset comprising pre-treatment tumor samples from eight patients with EGFR-mutant lung cancer treated with the tyrosine kinase inhibitor Osimertinib^[38]^. All patients had relapsed after initial response and were classified into two groups: short-term recurrence (< 8.6 months; *n* = 4) and long-term recurrence (≥ 12 months; *n* = 4). Each pre-treatment sample was scored using the A549 global RAC signature and the three supercluster signatures. We found that patients with short-term relapse exhibited higher resistance scores across all signatures (*n* = 8, Figure 3C). Statistical significance was achieved in one case, likely due to the small sample size. These findings suggest the presence of pre-existing resistance mechanisms in short-term responders.

We then assessed whether the supercluster signatures could predict therapy response across multiple cancer types and drug regimens. We analyzed pre-treatment bulk RNA sequencing and microarray datasets spanning six cancer types and seven drug treatments, in which patients were categorized as responders or non-responders^[39]^ [Supplementary Table 3]. On average, non-responders exhibited higher supercluster signature scores across all cohorts, although the difference did not reach statistical significance (one-sided *t*-test, *P*-value = 0.08; Figure 3D).

Finally, we examined whether supercluster signatures are associated with the progression of premalignant lesions. To this end, we analyzed a dataset from the HTAN database^[40,41]^ that includes 97 premalignant breast lesion samples, of which 31 progressed to cancer and 66 did not. We scored each sample using the MCF7 global RAC signature and the three supercluster signatures. Samples from the progression group showed higher scores, with statistical significance observed only for the supercluster 2 signature [Figure 3E]. Consistently, we also found that genes differentially expressed in the progression group were significantly enriched in the supercluster 2 signature [Supplementary Figure 2C]. These results suggest that resistance-associated signatures are linked, to some degree, with malignant transformation.

### Supercluster resistance mechanisms are evolutionarily conserved

**Figure 4:**
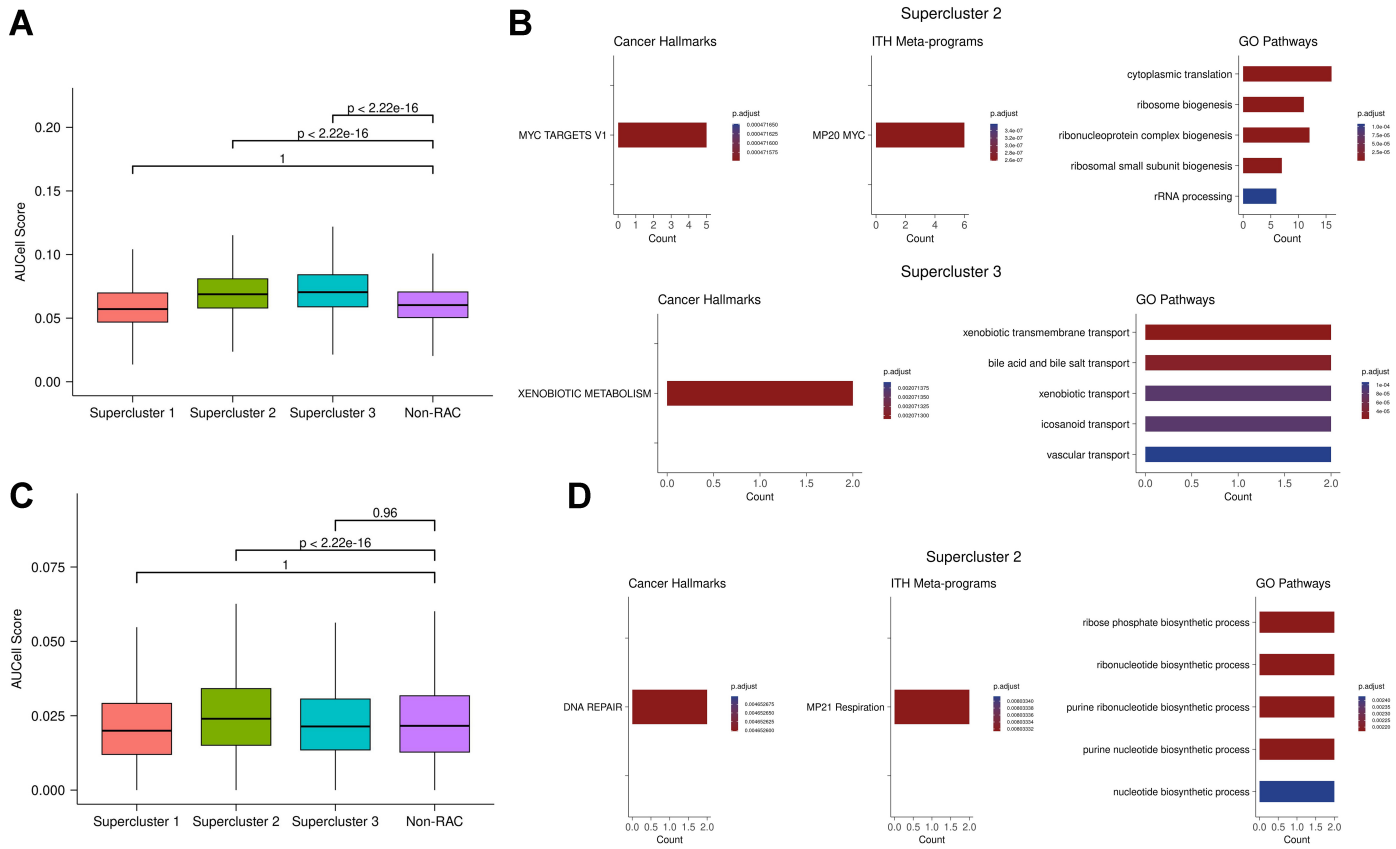
(A) *C. auris* antifungal resistance gene signature scores in human resistance and non-resistance clusters. Each distribution shows scores for individual cells in each supercluster or non-RAC cluster across three cell lines; (B) Pathway enrichment analysis of shared genes between *C. auris* resistance orthologs and superclusters 2 and 3. “Count” indicates the number of overlapping genes between the gene set of interest and the enriched pathway; (C) *E. coli* antimicrobial resistance gene signature scores in human resistance and non-resistance clusters. Each distribution shows scores for individual cells in each supercluster or non-RAC cluster across three cell lines; (D) Pathway enrichment analysis of shared genes between *E. coli* resistance orthologs and supercluster 3. RAC: Resistance-activated cluster.

Having identified early cellular responses broadly associated with resistance, we hypothesized that these resistance-related transcriptional responses reflect an inherent cellular reaction to chemically induced stress and are therefore evolutionarily conserved across species. To test this, we retrieved a gene signature comprising genes upregulated in the antifungal-resistant yeast *C. auris*^[13]^ and mapped them to their human orthologs^[26]^ [Supplementary Table 4]. We then scored all cells in the early-response dataset and compared score distributions between supercluster cells and non-RAC cells. In superclusters 2 and 3, cells exhibited significantly higher scores for the *C. auris* resistance ortholog signature compared to non-RAC cells, with Cohen’s d effect sizes of 0.58 and 0.70, respectively. This suggests that superclusters 2 and 3 may represent conserved drug response mechanisms shared between yeast and humans [Figure 4A].

To further investigate the underlying shared mechanisms, we performed functional enrichment analysis on genes common to the *C. auris* resistance ortholog set and the genes upregulated in superclusters 2 and 3 [Figure 4B]. This analysis revealed enrichment for MYC activity and ribosome biogenesis, suggesting that these processes are evolutionarily conserved responses to chemical stress and may contribute to resistance. The observed increase in MYC activity and ribosome biogenesis aligns with previous studies showing that MYC is a key regulator of ribosome biogenesis^[42,43]^, and both processes are implicated in drug resistance in yeast and human cancers^[31,44,45]^. Additionally, pathways enriched in the overlap between *C. auris* orthologs and supercluster 3 include xenobiotic metabolism and various transport mechanisms. Notably, genes such as ABCC2 and ABCC3 (orthologous to the *C. auris* gene B9J08_002146) contribute to this enrichment. These genes encode ATP-binding cassette (ABC) transporters, which are known to drive multidrug resistance (MDR) in both yeast and human cancer^[46,47]^. Taken together, these findings suggest that resistance mechanisms conserved between *C. auris* and human cancer involve MYC, its downstream regulatory processes, and drug efflux pumps.

To explore conservation of resistance mechanisms across additional species, we analyzed expression data from drug-treated *E. coli* strains. We identified genes upregulated in resistant versus drug-naive conditions across nine antimicrobial treatments and compiled a consensus antimicrobial resistance gene set [Supplementary Table 4]. These *E. coli* resistance genes were then mapped to their human orthologs, and we scored all human cells for the resulting gene signature. In supercluster 2, resistance scores were significantly higher than in non-RAC cells, with an effect size of 0.16 [Figure 4C].

We then performed pathway enrichment on genes shared between the *E. coli* resistance ortholog set and those upregulated in supercluster 2 [Figure 4D]. The enriched pathways suggest that DNA repair is a conserved resistance mechanism between *E. coli* and human cancer. This is consistent with previous studies demonstrating that enhanced DNA repair contributes to resistance in both contexts^[48,49]^. Overall, these analyses support the hypothesis that superclusters 2 and 3 represent evolutionarily conserved mechanisms of drug resistance. Specifically, superclusters 2 and 3 appear to reflect shared resistance pathways between humans and *C. auris*, involving the proto-oncogene MYC, its downstream targets such as ribosome biogenesis, and drug efflux systems. Additionally, supercluster 2 exhibits conserved resistance mechanisms between humans and *E. coli*, notably involving enhanced DNA repair.

## DISCUSSION

In this study, we comprehensively assessed the extent to which transcriptional programs that characterize drug resistance in cancer cells are either pre-existing prior to treatment or emerge early in response to therapy. These early programs may represent intrinsic cellular responses to cytotoxic stress, some of which form the basis for long-term resistance. We found that transcriptional programs typically observed in established resistance - such as EMT^[27,50]^, MYC signaling^[31,32]^, and oxidative phosphorylation^[33]^ - are already detectable just 24 h after drug treatment. Moreover, these early manifestations are not limited to a single cancer type; they are evident across multiple cancer cell lines. Notably, aspects of these responses show potential evolutionary conservation in bacteria and yeast, suggesting that such intrinsic resistance programs may represent a fundamental cellular survival mechanism conserved across species. Previous studies have shown that genes with high expression variability often emerge as differentially expressed across unrelated experiments and are enriched for stress-response functions^[51,52]^. Ultimately, causality must be established through targeted experimental approaches. Using a whole-genome CRISPR knockout screen, we validated that disrupting genes associated with early resistance states increases drug sensitivity in a manner consistent with the known mechanisms of action of the respective drugs. Further validation, including longitudinal lineage tracing under perturbation, could strengthen these findings and confirm causal roles for the identified gene signatures. Importantly, these early response signatures also show prognostic value in premalignant states and overall survival, suggesting that they may reflect cell states inherently resistant to diverse stressors that cells have faced throughout evolution.

It is well established that many cancer treatments compromise immune infection, increasing infection risk in patients with advanced disease^[53,54]^. Our finding that components of the early drug response are conserved across species raises the possibility of developing therapies that simultaneously target cancer resistance and pathogen resistance. By focusing on conserved resistance mechanisms, such therapies could improve treatment efficacy while lowering infection-related mortality^[54]^.

Consistent with previous reports^[8,9]^, our analyses reveal distinct modes of resistance. These resistance-associated transcriptional states, conserved across cancer types, differ in their cell cycle status and are associated with specific drug classes [Figure 2]. Remarkably, even in pre-treatment tumor transcriptomes, these early response signatures correlate with relapse timing, overall survival, therapy response, and tumor progression across a range of cancers and therapies [Figure 3]. Since the data used for these predictions were obtained at diagnosis, before chemotherapy, it is likely that the resistant states were already present and subsequently selected for during treatment. Conversely, other transcriptional states appear only after treatment, suggesting they may represent adaptive responses [Figure 1C and Supplementary Figure 1B]. Our findings corroborate recent reports on DTP cells, which are rare, slow-cycling cells that transiently persist post-treatment and can resume proliferation upon drug withdrawal, becoming re-sensitized to the original therapy^[6,55]^. However, whether DTPs are pre-existing or arise via therapy-induced reprogramming remains unclear^[7]^, and this question could be best addressed through lineage tracing. Additionally, rare pre-existing resistant populations might evade detection in single-cell profiling due to limited sensitivity for capturing low-frequency states, leading them to appear as emergent after treatment.

Although we observed consistent expression of resistance signatures across several cancer types, their robustness could be improved. Our initial resistance signature was derived from only six datasets, and the early response signature from just three cell lines. Expanding this analysis to include additional cancer types could enhance the generalizability of these signatures and potentially uncover resistance mechanisms not captured here. Tumor-specific variations in the tumor microenvironment - particularly *in vivo* or in organoid models - likely contribute to additional resistance programs absent in our current analysis. Moreover, many of our conclusions are based on correlational signature scoring; hence, future CRISPR-based functional studies will be needed to validate the biological causality of these associations.

Our drug response dataset includes a wide range of drugs and classes, lending broad relevance to the identified resistance-associated signatures^[11]^. One well-known mechanism underlying MDR involves ABC transporters. We found that all three major gene signatures - cell type-specific global RAC, supercluster 1, and supercluster 3 - include ABC transporter genes (e.g., ABCA1, ABCA12, ABCB1, ABCC2, ABCC3, ABCC13, and ABCG1), indicating their potential role in resistance across cell lines. While P-glycoprotein 1 (Pgp, ABCB1) has been shown to be inducible by drug treatment^[56]^, the extent of its involvement in the resistance observed in our study remains to be determined.

In conclusion, we identified multiple transcriptional states associated with non-specific drug resistance mechanisms across various cancer types and even across species such as *E. coli* and *C. auris*. The gene signatures derived from these resistant states offer promising avenues for further investigation^[57]^. Although more research is needed to fully elucidate the mechanisms of resistance and strategies for their inhibition, our findings contribute to a growing understanding of the molecular features of early resistant transcriptional states. These insights may ultimately inform the development of novel adjuvant therapies to prevent or overcome resistance.

## DECLARATIONS

### Availability of data and materials

All data needed to evaluate the conclusions in the paper are present in the paper and/or the Supplementary Materials. Public datasets used in this study are available in the referenced papers. Source code and analysis scripts are available on GitHub: (https://github.com/coleruoff/drug_treatment).

### Copyright

© The Author(s) 2025.
