# Supplementary material for: Resistance signatures manifested in early drug response across cancer types and species
Source: Cancer Drug Resist. 2025 Aug 26;8:44. doi: 10.20517/cdr.2025.112 (PMC12462399; doi:10.20517/cdr.2025.112)
Supplement: Supplementary file 1 [file cdr-8-44-SupplementaryMaterials.zip › cdr80112-SupplementaryMaterials.pdf]

Supplementary Materials

Resistance signatures manifested in early drug response across cancer types and species

Cole Ruoff[1], Allison Mitchell[2], Priya Mondal[2], Vishaka Gopalan[1], Arashdeep Singh[1], Michael Gottesman[2], Sridhar Hannenhalli[1]

[1]Cancer Data Science Lab, Center for Cancer Research, National Cancer Institute, National Institutes of Health, Bethesda, MD 20892, USA.
[2]Laboratory of Cell Biology, Center for Cancer Research, National Cancer Institute, National Institutes of Health, Bethesda, MD 20892, USA.

Correspondence to: Dr. Sridhar Hannenhalli, Cancer Data Science Lab, Center for Cancer Research, National Cancer Institute, National Institutes of Health, Room 200. NIHBC 15G1, 9000 Rockville Pike, Bethesda, MD 20892, USA. E-mail: Sridhar.hannenhalli@nih.gov

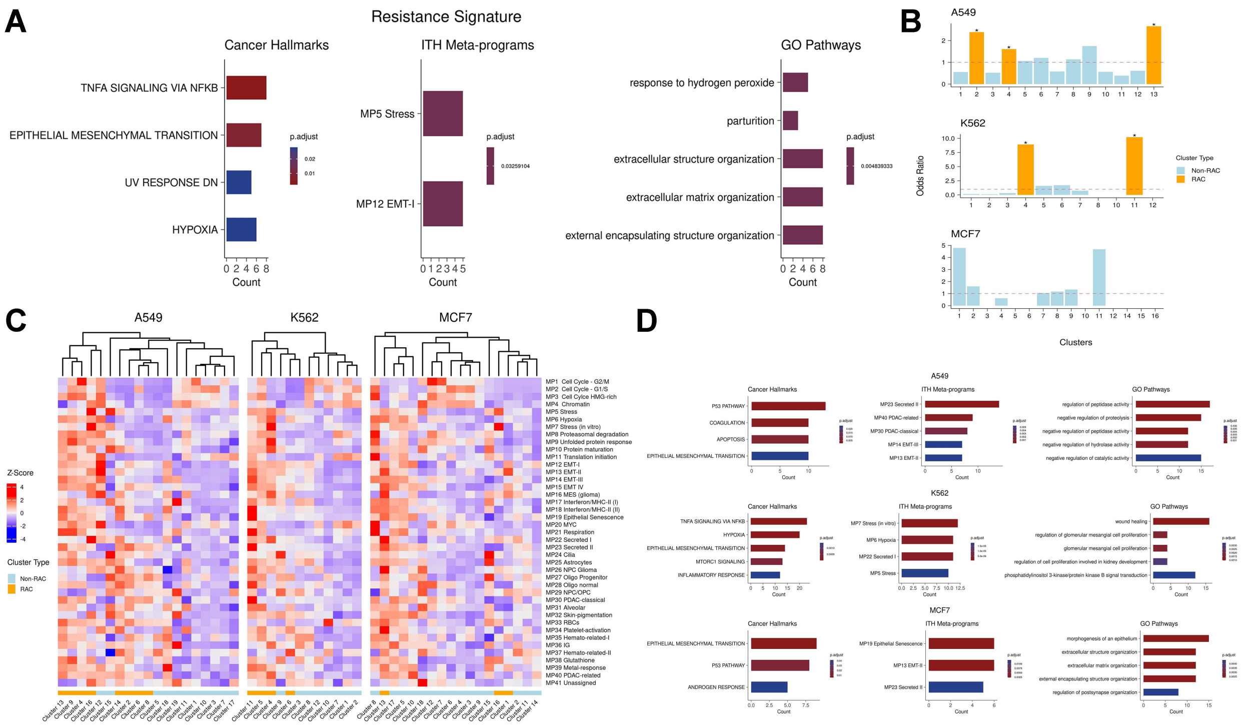

Supplementary Figure 1. (A) Pathway enrichment of drug resistance signature. Each plot shows the overrepresentation of cancer hallmarks, ITH meta-programs, and GO pathways respectively. (B) Pre-treatment resistant active to inactive cell odds ratio of each cluster in each cell line. The y axis represents the odds ratio value and each bar represents a transcriptional cluster. Any cluster with an odds ratio greater than 1.5 and a p-value less than 0.05 is considered a Resistance Activated Cluster (RAC) and is colored orange, while the remaining clusters are non-RACs and are colored blue. (C) Intratumor Heterogeneity Meta-program mean AUCell score for all clusters in each cell line. The rows represent the different ITH MP genesets and the columns represent each transcriptional cluster, separately for each cell line. The colors in the bottom row signifies which cluster is a Resistance Activated Cluster (RAC) (orange = RAC, blue = Non-RAC). (D) Enrichment of the global RAC signature in each cell line. The columns from left to right represent cancer hallmarks, ITH meta-programs, and GO pathways respectively. Within each column, the rows represent the three cell lines. Count values indicate the number of shared genes between the global RAC signature and the enriched pathway.

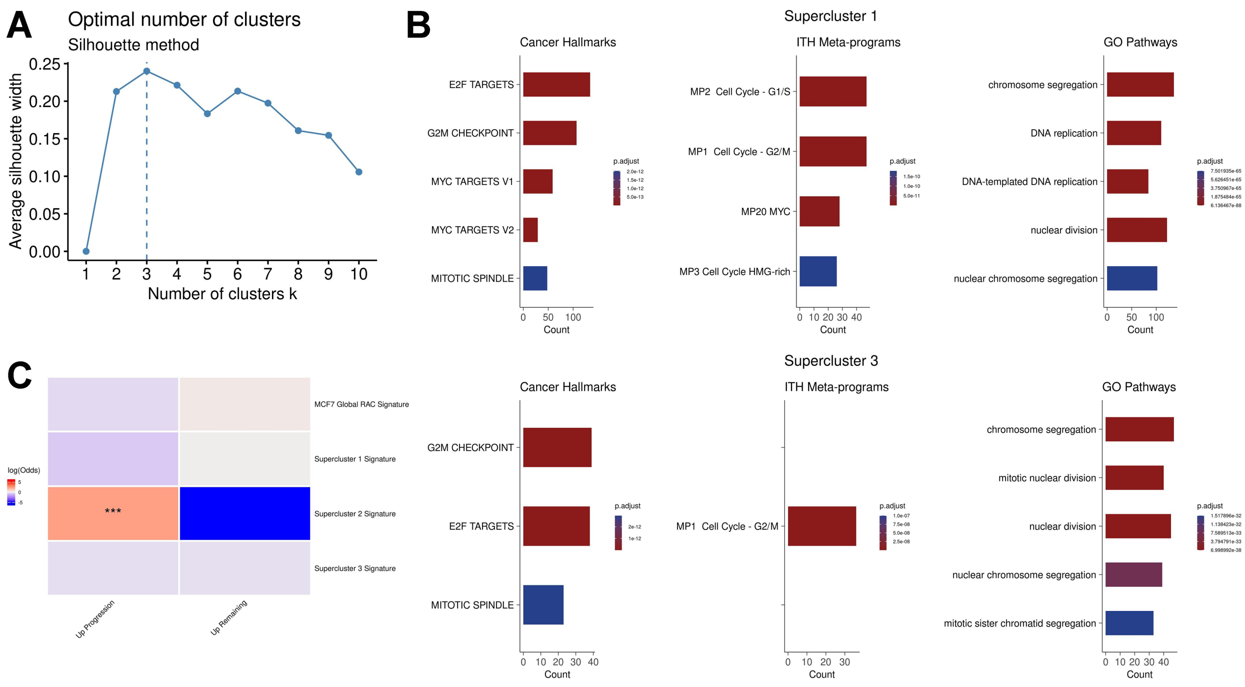

Supplementary Figure 2. (A) Optimal number of superclusters. Line plot showing the silhouette score for each number of superclusters. Vertical line drawn at maximum value indicating the optimal number of superclusters. (B) Supercluster Downregulated Signatures Enrichment. The columns from left to right represent cancer hallmarks, ITH meta-programs, and GO pathways respectively. Within each column, the rows represent the three superclusters. Count values indicate the number of shared genes between the supercluster downregulated signature and the enriched pathway. (C) Shared progression-associated genes. Displays the log odds ratio of overrepresentation test observing the overlapping genes between the four resistance signatures and progression-associated genes.

Supplementary Details:

1. **Global RAC signature enrichment:**

As a complement to the hallmark and ITH meta-program analysis, in each cell line, we determined the differentially expressed genes between all RAC cells and all non-RAC cells (Table S1; Methods), and assessed these genes for enrichment of Cancer Hallmarks, ITH meta-programs, and various GO biological processes (Methods; Figure S1D). Across all three cell lines, we observed enrichment of drug resistance-associated pathways such as EMT which previously has been shown to contribute to the development of drug resistance. Other drug resistance-associated pathways enriched in the RAC signatures include TNFa and hypoxia, both associated with drug resistance in cancer. The enrichment of these pathways further suggests that RAC cells may represent the early stages of drug resistant cell states.

2. **Supercluster downregulated gene signatures enrichment:**

Repeating functional enrichment analysis of the downregulated gene sets in each supercluster resulted in very few pathways. Of the three superclusers, only supercluster 1 and 3 displayed any significant enrichment of functional pathways (Figure S2B). Among these pathways were mostly cell cycling pathways further indicating that supercluster 1 and 3 downregulate cycling pathways and exhibit a more queiscent state, a common feature of resistant states.
